# Mitochondriotropic and Cardioprotective Effects of Triphenylphosphonium-Conjugated Derivatives of the Diterpenoid Isosteviol

**DOI:** 10.3390/ijms18102060

**Published:** 2017-09-26

**Authors:** Lara Testai, Irina Strobykina, Victor V. Semenov, Marina Semenova, Eleonora Da Pozzo, Alma Martelli, Valentina Citi, Claudia Martini, Maria C. Breschi, Vladimir E. Kataev, Vincenzo Calderone

**Affiliations:** 1 Department of Pharmacy, University of Pisa, Bonanno Street, 6, 56120 Pisa, Italy; eleonora.dapozzo@unipi.it (E.D.P.); alma.martelli@unipi.it (A.M.); valentina.citi@for.unipi.it (V.C.); claudia.martini@unipi.it (C.M.); maria.breschi@unipi.it (M.C.B.); vincenzo.calderone@unipi.it (V.C.); 2 A. E. Arbuzov Institute of Organic and Physical Chemistry, Kazan Scientific Center of Russian Academy of Sciences, Arbuzov Street, 8, 420088 Kazan, Russia; strobykina@iopc.ru (I.S.); kataev@iopc.ru (V.E.K.); 3 N. D. Zelinsky Institute of Organic Chemistry, Russian Academy of Sciences, Leninsky Prospect, 47, 119991 Moscow, Russia; vs@zelinsky.ru; 4 N. K. Kol’tsov Institute of Developmental Biology, Russian Academy of Sciences, Vavilov Street, 26, 119334 Moscow, Russia; ms@chemical-block.com

**Keywords:** mitochondria, *Stevia rebaudiana* Bertoni, tetraphenylphosphonium, isosteviol, cardioprotection

## Abstract

Mitochondria play a crucial role in the cell fate; in particular, reducing the accumulation of calcium in the mitochondrial matrix offers cardioprotection. This affect is achieved by a mild depolarization of the mitochondrial membrane potential, which prevents the assembly and opening of the mitochondrial permeability transition pore. For this reason, mitochondria are an attractive target for pharmacological interventions that prevent ischaemia/reperfusion injury. Isosteviol is a diterpenoid created from the acid hydrolysis of *Stevia rebaudiana* Bertoni (fam. Asteraceae) glycosides that has shown protective effects against ischaemia/reperfusion injury, which are likely mediated through the activation of mitochondrial adenosine tri-phosphate (ATP)-sensitive potassium (mitoKATP) channels. Some triphenylphosphonium (triPP)-conjugated derivatives of isosteviol have been developed, and to evaluate the possible pharmacological benefits that result from these synthetic modifications, in this study, the mitochondriotropic properties of isosteviol and several triPP-conjugates were investigated in rat cardiac mitochondria and in the rat heart cell line H9c2. This study’s main findings highlight the ability of isosteviol to depolarize the mitochondrial membrane potential and reduce calcium uptake by the mitochondria, which are typical functions of mitochondrial potassium channel openings. Moreover, triPP-conjugated derivatives showed a similar behavior to isosteviol but at lower concentrations, indicative of their improved uptake into the mitochondrial matrix. Finally, the cardioprotective property of a selected triPP-conjugated derivative was demonstrated in an in vivo model of acute myocardial infarct.

## 1. Introduction

Mitochondria are an attractive target for pharmacological interventions that prevent ischaemia/reperfusion (I/R) injuries. Mild depolarization of the mitochondrial membrane potential reduces calcium uptake into the matrix and consequently prevents the assembly and opening of the mitochondrial permeability transition pore (MPTP) [1]. This beneficial effect can result from the actions of multiple mitochondrial targets, such as kinase proteins, oxidative phosphorylation, ion channels, and, in particular, potassium channels. According to the literature, diverse targets are often involved in I/R injury protection, highlighting the complexity of the mitochondrial mechanisms of cardioprotection; however, several types of potassium channels are localized on the inner membrane of the mitochondria—for example, the calcium-dependent (mitoBK), ATP-sensitive (mitoKATP), and recently voltage-operated (mitoKv7.4) channels have been considered exciting targets for myocardial protection [1,2].

Isosteviol is a diterpenoid that is easily obtained by the acid hydrolysis of *Stevia rebaudiana* Bertoni (fam. Asteraceae) glycosides, which are used for the production of non-caloric sugar substitutes [3]. Several studies have indicated that isosteviol possesses a variety of biological activities including anti-hypertensive [4,5,6], anti-hyperglycemic [7], antioxidant, anti-inflammatory, and antitumor effects [8,9]; in addition, it relieves I/R injury in rat brains [10] and in rodent hearts [11,12]. Xu and colleagues demonstrated for the first time that isosteviol produces cardioprotective effects in rats subjected to myocardial I/R injury. Indeed, isosteviol administered by intravenous (i..v.) for 10 min before coronary occlusion protected the myocardium (decreased levels of lactate dehydrogenase (LDH) and creatine kinase (CK)), reduced the myocardial infarct size, improved cardiac performance (increased dp/dtmax, left ventricular systolic pressure (LVSP), and left ventricular developed pressure (LVDevP)), and stabilized the electrophysiological properties of the heart (decreased occurrence of ventricular tachycardia (VT) and ventricular fibrillation (VF)). The authors suggested that at least some of the observed beneficial effects of isosteviol can be attributed to stimulation of the mitoKATP channel [11]. Similar conclusions were reported by Xu et al., 2007 in an ex vivo model, and the involvement of the mitoKATP channel was also hypothesized since a selective blocker of mitoKATP channels, 5-hydroxydecanoic acid (5HD), antagonized its protective effects [12].

However, the involvement of alternative pathways is suggested in isosteviol-mediated myocardial protection. Recently, Fan and colleagues observed that when isosteviol was administered alone, it failed to produce any effects, whereas it increased the pinacidil-induced activation of sarcKATP channels and potentiated the diazoxide-mediated oxidation of flavoproteins in mitochondria, suggesting the engagement of mitoKATP channels [13,14].

The above literature suggests a key role for mitoKATP channels in the isosteviol cardioprotective profile, which may be an exciting strategy for effectively driving diterpene into the mitochondria to improve its pharmacokinetic profile and, consequently, its pharmacological effects.

Recently, isosteviol derivatives with carboxylic groups linked to the cationic triphenylphosphonium (triPP) moiety by different linkers have been synthesized [15]. Conjugation to the triPP moiety ensures high accumulation in the mitochondrial matrix due to the negative relative voltage (about −180 mV), according to Nernst’s law. Indeed, there are many examples of the triPP moiety being successfully used as a carrier for the preferential delivery of drugs into the mitochondria [16]. Accordingly, triPP-conjugates of isosteviol exhibited pronounced antimitotic effects in the sea urchin embryo model, indicative of mitochondrial uptake; moreover, a preliminary structure–activity relationship study revealed the essential role of the triPP moiety for the isosteviol effect since the chemical structure and the length of the linker had only limited impact on the biological activity of these derivatives [15].

In this study, the mitochondriotropic properties of isosteviol and several triPP-conjugates were investigated on rat cardiac mitochondria and on H9c2 cells in order to evaluate possible pharmacological benefits resulting from such synthetic modifications.

## 2. Results

### 2.1. Mitochondriotropic Effects of Isosteviol

**Figure 1:**
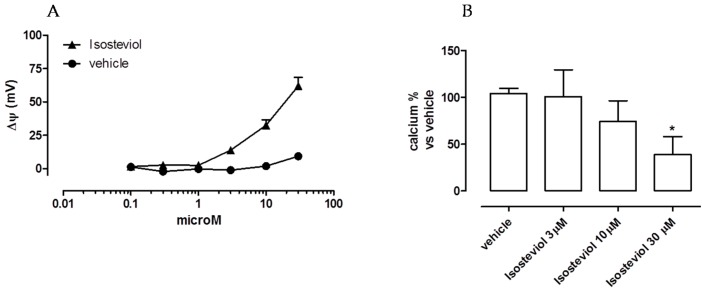
Changes of mitochondrial membrane potential (**A**) and of calcium uptake into mitochondrial matrix (**B**), potentiometrically measured, following addition of isosteviol at cardiac mitochondria suspensions (1 mg/mL). Asterisks indicate significant differences vs. vehicle (* *p* < 0.05).

All isolated heart mitochondria had a membrane potential lower than −170 mV. The addition of cumulatively increasing concentrations of isosteviol (0.1–30 μM) to the suspension of isolated heart mitochondria (1 mg/mL) caused a concentration-dependent Δ*ψ* depolarization. The vehicle was almost completely ineffective at depolarizing the mitochondria (Figure 1A).

Moreover, the addition of calcium-free mitochondria into calcium-rich medium (100 μM) nearly depleted this ion (108 ± 7 μM, *n* = 3) from the medium, indicating an avid uptake of calcium into the mitochondrial matrix. The addition of isosteviol (3, 10, and 30 μM) to the medium caused a concentration-dependent attenuation of calcium ion accumulation in the mitochondria (Figure 1B).

### 2.2. Mitochondriotropic Effects of Tripp-Conjugated Isosteviol Derivatives

Due to the inevitable interference of triPP-conjugated derivatives with the tetraPP-sensitive electrode, the potentiometric approach was not suitable for the measurement of Δ*ψ* changes in this treatment group. Therefore, a spectrofluorimetric method was applied, despite compromised sensitivity of this method compared to that of the potentiometric approach.

As expected, the well-known uncoupling agent CCCP (0.01–1 μM) produced a marked and concentration-dependent depolarizing effect, leading to the complete abolition of the Δ*ψ* value (Figure S1).

**Figure 2:**
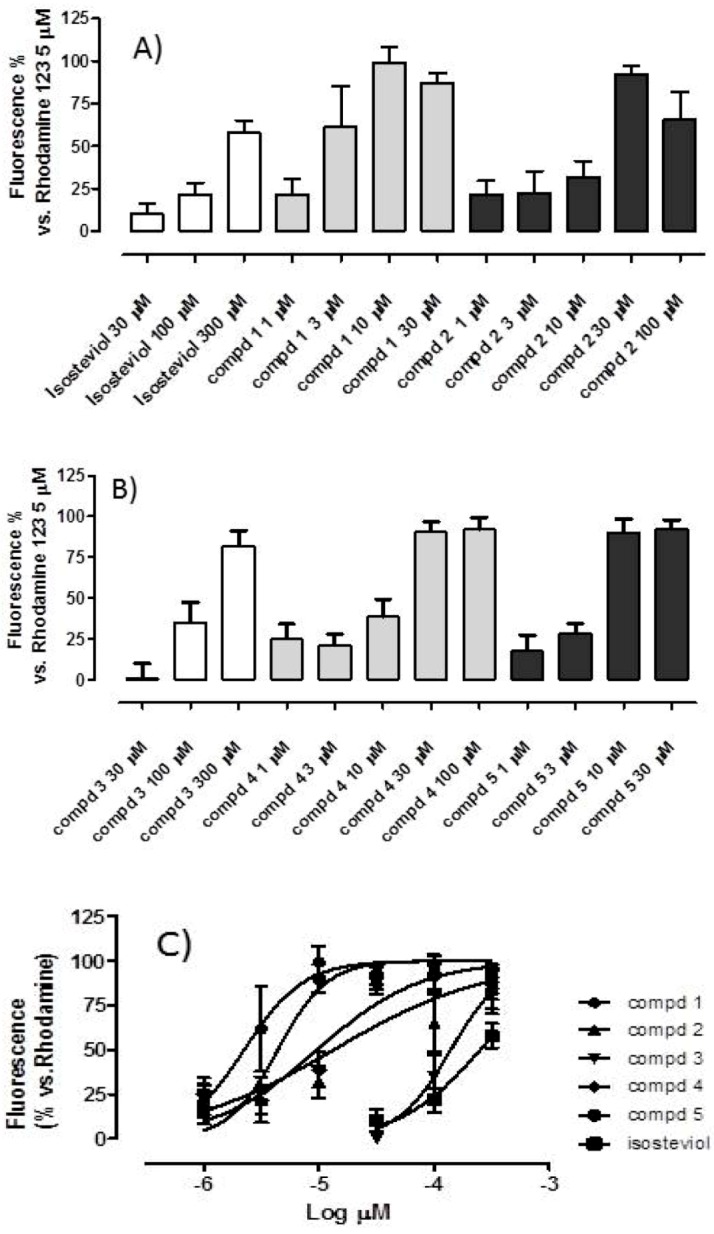
Changes of mitochondrial membrane potential (spectrofluorimetrically measured) following addition at cardiac mitochondria suspension (0.5 mg/mL) (**A**,**B**). Increase (%) in the rhodamine123-induced fluorescence after cumulative addition of isosteviol and compounds 1–5 (**C**).

Isosteviol influenced the Δ*ψ* only at high concentrations (300 μM), producing a fluorescence signal up to 58 ± 7% of the signal obtained with rhodamine 123 alone (Table 1, Figure 2A–C).

In contrast, all tested triPP-conjugated compounds produced strong concentration-dependent depolarizing effects.

Notably, the isosteviol derivatives, especially compounds **1** and **5**, which have the longest alkyl chains, showed an improved potency compared with that of the parent isosteviol (Table 1 and Figure 2A–C).

### 2.3. Uptake of Compounds ***1*** and ***5*** into the Mitochondrial Matrix

**Figure 3:**
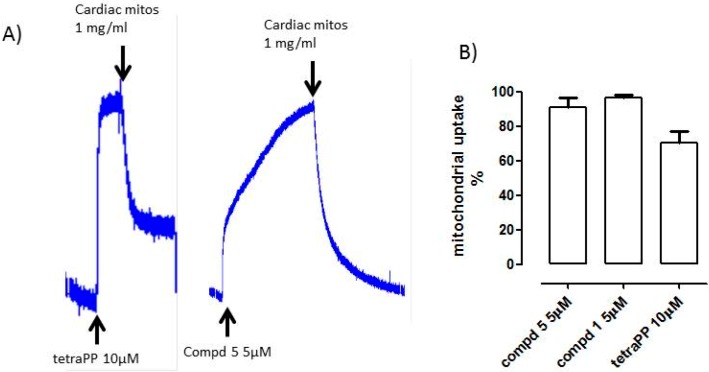
(**A**) Exemplificative tracing of mitochondrial uptake of tetraPP 10 microM and of the most potency triPP conjugated derivatives (compd **1** and **5**); (**B**) Percentage of cardiac mitochondrial uptake of tetraPP and triPP-conjugated derivatives of isosteviol (cmpd 1 and 5).

When added to the medium, both the triPP-conjugated compounds **1** and **5** and tetraPP were immediately detected by the tetraPP-sensitive electrode. TetraPP accumulated according to Nernst’s law, indicative of an optimal potential value (about −180 mV). The introduction of cardiac mitochondria (1 mg/mL) to the suspension medium containing compound **1** or **5** caused a disappearance (downward deflection of the signal) of compounds **1** or **5** in the suspension medium, due to their uptake into the mitochondrial matrix (Figure 3B). Notably, both triPP-conjugated derivatives were completely taken into the matrix, to an even greater extent than tetraPP. A representative experiment is shown in Figure 3A.

### 2.4. Effects of Isosteviol and Compound ***5*** Treatment on the Δψ of H9c2 Cells

Mild depolarization of the mitochondrial inner membrane potential attenuates mitochondrial reactive oxygen species generation and is defined as positive depolarization.

**Figure 4:**
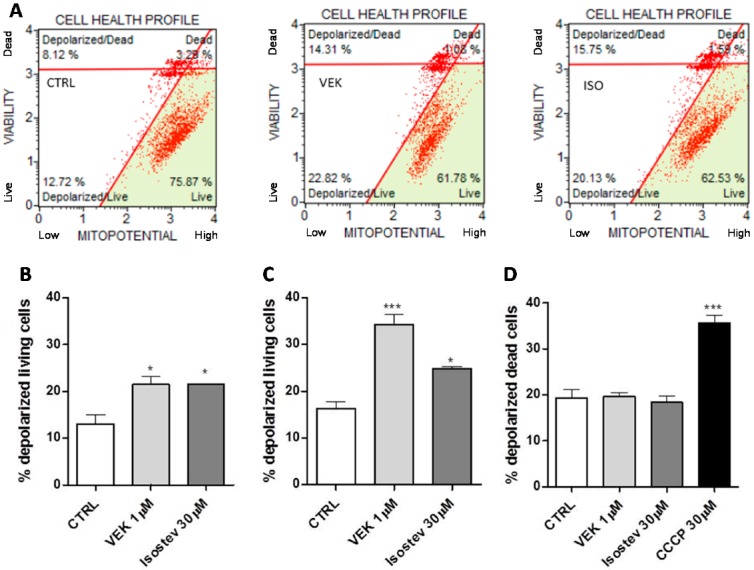
Change in mitochondrial Δ*ψ* related to compound **5** and isosteviol cell treatments. H9c2 cells were challenged with compound **5**, isosteviol, and CCCP for 2.5 and 5 h, then the mitochondrial Δ*ψ* was assessed by a cytofluorimetric technique. In panel (**A**), representative cytofluorimetric population profiles are reported (2.5 h challenge). The percentage of depolarized living cells after 2.5 and 5 h of drug treatments are shown in panels (**B**,**C**), respectively. The percentage of depolarized dead cells following 5 h (**D**) are also reported. Asterisks indicate significant differences vs. vehicle (* *p* < 0.05, *** *p* < 0.001).

To monitor changes in the mitochondrial Δ*ψ*, drug-treated H9c2 cells were incubated with the MitoPotential Reagent, a cationic lipophilic dye that accumulates within the inner membrane of intact mitochondria resulting in high fluorescence emissions. Cells with depolarized mitochondria have a decrease in fluorescence. Then, 7-AAD was also added to the cells as an indicator of cell membrane structural integrity and cell death, since it is excluded from live cells. Four populations of cells can be distinguished by the assay: live cells with intact mitochondrial membranes, live cells with depolarized mitochondrial membranes, dead cells with depolarized mitochondrial membranes, and dead cells with intact mitochondrial membranes (Figure 4A). Positive depolarization is represented by live cells with mildly depolarized mitochondrial membranes.

As reported in Figure 4, after 2.5 h of treatment (B), both compound **5** and isosteviol caused a mild increase in the depolarization of living cells compared to that of the control cells (*p* < 0.05). According to the best efficiency of compound **5** entering the mitochondria, the longer drug incubation time (5 h of treatments, Figure 4C,D) increased the percentage of depolarized living compound **5**-treated cells, which was statistically significant compared to that of the control cells (*p* < 0.001, Figure 4C). Interestingly, neither compound **5** nor isosteviol caused an increase in the percentage of depolarized dead cells after 5 h of treatment; however, cells treated with the uncoupling agent CCCP showed a highly statistically significant increase in the percentage of depolarized dead cells compared to that of the control cells (*p* < 0.001, Figure 4D).

### 2.5. Cardioprotective Effects Observed with Isosteviol and Compound ***5***

**Figure 5:**
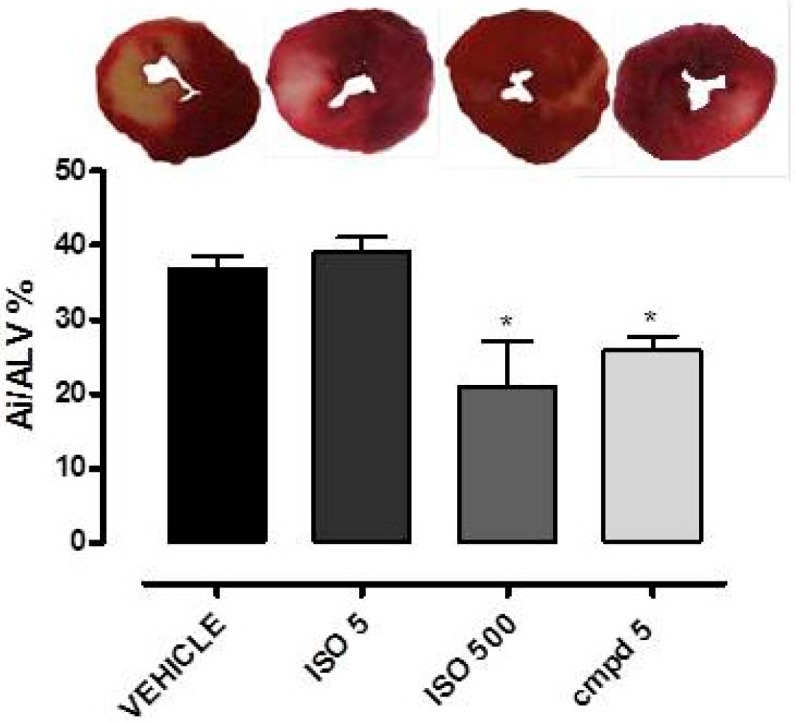
Morphometric quantification of ischaemia/reperfusion (I/R)-induced injury observed in ventricular slices of rat hearts, after acute myocardial infarction in vivo, after treatment with VEHICLE, ISO 500, ISO 5, or compound **5**. Asterisks indicate significant differences vs. vehicle (* *p* < 0.05).

In the SHAM group, no significant evidence of damage was observed (data not shown), whereas the acute infarct protocol led, in the VEHICLE group, to a marked myocardium damage (Ai/ALV = 37 ± 2%). In the ISO 500 group, a significant reduction of the size of damaged areas was observed (Ai/ALV = 21 ± 6%). In contrast, the cardiac protection was lost in the group ISO 5, which was treated with a 100-fold lower dose of isosteviol (Ai/ALV = 39 ± 2%). Noteworthy, the pharmacological treatment with compound **5** at the dose of 12.1 μg/Kg (equimolar to isosteviol 5 μg/Kg) produced a significant reduction of the ischaemic areas (Ai/ALV = 26 ± 2%), which was similar to that observed in the ISO 500 group (Figure 5).

## 3. Discussion

In conditions of cellular stress, mitochondria play a crucial role in cell fate since they can decide when and how a cell will die; they are considered an especially key component in cardiomyocyte dysfunction and cell death during I/R injury. It is widely accepted that reducing calcium overload in the mitochondrial matrix is essential for cardioprotection. A reduction in calcium uptake can be achieved by mild depolarization of the mitochondrial membrane. A modest depolarization prevents the entry of high amounts of calcium into mitochondria [1], thereby reducing the probability that the MPTP will form and open, and preserving cell viability during reperfusion. Thus, mitochondria are considered a promising target for modern cardioprotective strategies.

In the recent years, compounds from the plant kingdom—particularly the flavonoid class that contains interesting examples of natural compounds endowed with cardioprotective mitochondria-targeting effects—have gained attention for their therapeutic potential [17,18]. Other plant-derived compounds, such as the well-known sweetener steviol glycoside (rebaudiosides), have beneficial effects on the cardiovascular system [19]. In contrast to sugar, rebaudiosides (specially the glycoside stevioside) demonstrate anti-hyperglycemic, insulinotropic, and glucagonostatic effects [20,21]; moreover, isosteviol, a stevioside derivative, has protective effects against I/R damage in the hearts and brains of rats [10,11,12,13].

Although its mechanism of action is not fully known, the cardioprotective effect is clear. In this context, the first aim of this study was to demonstrate the role of mitochondria in the mechanism of action of isosteviol.

Indeed, when isosteviol was cumulatively administered to a mitochondrial suspension, concentration-dependent depolarization of the mitochondria occurred (Figure 1A), and this compound was able to influence the movement of calcium ions, reducing the entry of calcium into the mitochondrial matrix (Figure 1B).

Moreover, the use of novel drugs that are able to selectively accumulate in the mitochondria, avoiding off-target effects on other cellular components, is an exciting, novel strategy for providing protection against myocardial infarction. Accordingly, Murphy and colleagues [16] first developed the chemical strategy of conjugating a triPP moiety to an antioxidant agent to drive it into the mitochondrial matrix. This strategy has been used by other researchers to obtain selective mitochondrial delivery. In particular, Kataev’s group synthesized isosteviol derivatives with anti-mitotic activity [15].

The second objective of this study was focused on the characterization of the mitochondriotropic effects of the five novel isosteviol derivatives, which are derived from a diterpenoid skeleton coupled to a triPP moiety by polyethylene glycol or polymethylene linkers of different lengths (i.e., triPP-conjugates of isosteviol). To evaluate the depolarizing effects of these triPP-conjugates, the most sensitive potentiometric approach was not possible to use because of the inevitable interference with the tetraPP-sensitive electrode; therefore, we used a validated spectrofluorimetric method with rhodamine 123, a cationic probe whose fluorescence increases following the depolarization of mitochondria. All tested compounds produced a concentration-dependent depolarization compared with that of isosteviol (Figure 2A,B). An interesting structure-activity relationship was shown for compounds **1** and **5**, which both have polymethylene linkers; in fact, it is possible to correlate the depolarizing potency to the chain length connecting the isosteviol residue to the triPP moiety. The most potent derivative was compound **5**, which possesses the longest chain and thus is probably the most lipophilic compound of the series (Figure 2C).

The accumulation of the most potent compounds **1** and **5** (Figure 2C and Table 1) in the mitochondrial matrix was confirmed. This method revealed that compounds **1** and **5** were able to completely enter into the matrix, even more effectively than tetraPP alone (Figure 3), which is possibly due to the higher lipophilicity of these novel compounds than that of tetraPP.

Interestingly, in intact H9c2 cells, compound **5** was able to enter the mitochondrial matrix in a time-dependent manner and effectively produced a “beneficial” depolarization of the Δ*ψ* in living cells, which supports the protective role of this derivative in ischaemia-reperfusion insults. Moreover, compound **5** entered the mitochondria at lower concentrations than that required by isosteviol, based on the rationale by which the triPP-derivatives were synthetized.

However, the primary goal of this study is the development of innovative isosteviol-derived compounds endowed with cardioprotective effects. In an in vivo model of acute myocardial infarct in rats, isosteviol (ISO 500) showed significant cardioprotective effects, in good agreement with other scientific papers reporting the use of this dose of isosteviol [12]. When tested at a 100-fold lower dose, isosteviol did not show any significant cardioprotective effect. Instead, compound **5** at the equimolar dose of ISO 5 produced a significant reduction in the size of the myocardial damage, similar to that observed with higher dose of isosteviol (ISO 500), suggesting that the mitochondrial delivery ensured by the triPP-strategy led to a significant improvement of the cardioprotective effects.

Further experiments are required to verify the cardioprotection offered by these innovative compounds in myocardial I/R models, and their pharmacokinetic profile must be investigated to verify their entry into the mitochondria after administration in vivo.

## 4. Materials and Methods

### 4.1. Substances

**Figure 6:**
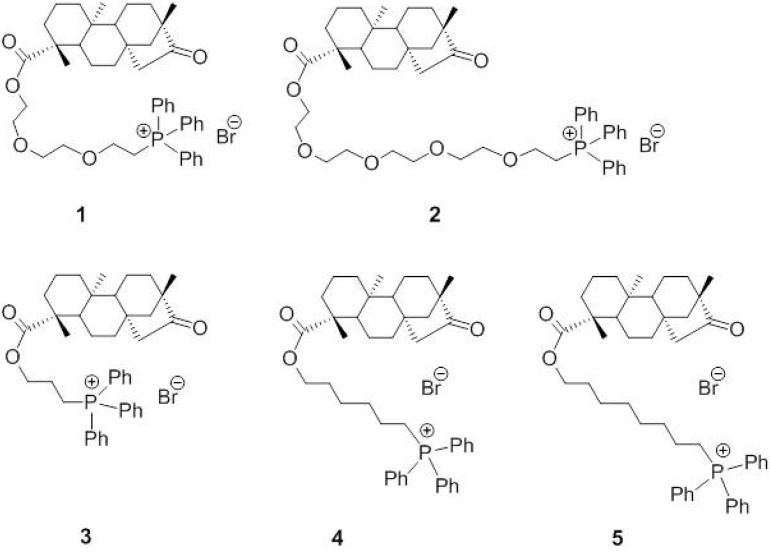
Chemical structure of triphenylphosphonium (triPP)-conjugated derivatives of isosteviol.

Sodium pentobarbital was purchased from Carlo Sessa in Milan, Italy. Isosteviol and the triPP-derivatives (compounds **1**–**5**; Figure 6) were synthesized according to the method reported previously by Strobykina et al. [15], dissolved (10^−2^ M) in pure dimethyl sulfoxide (DMSO), and further diluted in double-distilled water. Tetraphenylphosphonium chloride (tetraPP), rhodamine 123, oligomycin, carbonyl cyanide *m*-chlorophenylhydrazone (CCCP), and valinomycin were purchased from Sigma–Aldrich (St. Louis, MO, USA), dissolved in pure DMSO (100 mM), and further diluted in double-distilled water.

### 4.2. Experimental Procedures

All experimental procedures were carried out following the guidelines of the European Community Council Directive 2010/63 and were approved by the Ethical Committee for animal experimentation of Pisa University (Protocol. N. 37321, 22 October 2013). All animals were housed (two per cage) in a room with controlled temperature (23–25 °C), humidity (50%), and lighting (12 h light/dark cycle) and provided with food and water ad libitum. On the day of experiment, male rats (300–400 g of body weight) were euthanized with an overdose of sodium pentobarbital (100 mg/kg, i.p.) and blood samples were collected.

#### 4.2.1. Isolation Procedure of Mitochondria

Rat cardiac mitochondria were isolated by differential centrifugation according to the method of Chappel and Hansford [22], as previously published [23].

Briefly, the heart was removed immediately and placed into ice cold isolation buffer (composition: 250 mM sucrose, 5 mM Tris, and 1 mM ethylene-bis(oxyethylenenitrilo)tetraacetic acid (EGTA, pH 7.4, adjusted with HCl)). Atria were removed, and the ventricular tissues were finely minced with surgical scissors (approximately 2 mm^3^ pieces) and homogenized using an Ultra-Turrax homogenizer (20 mL of isolation buffer per heart).

Three homogenization cycles (20 s each) were performed on ice; then, the suspension was centrifuged at 1075× *g* for 3 min at 4 °C. The resulting supernatant was again centrifuged at 11,950× *g* for 10 min at 4 °C. The pellet, containing the mitochondrial fraction, was resuspended in isolation buffer (without EGTA) and centrifuged at 11,950× *g* for 10 min at 4 °C; this step was repeated once more.

The final mitochondrial pellet was resuspended in a minimal volume of 400 μL of isolation buffer (without EGTA). Mitochondria were stored on ice throughout processing and used within 2 h from isolation. Mitochondrial protein concentrations were determined using the Bradford reaction [24,25].

##### Measurements of the Effects of Isosteviol on Mitochondrial Ca^++^ Uptake

Changes of the medium (i.e., extra-mitochondrial) Ca^++^ concentration were continuously measured with a Ca^++^ selective mini-electrode coupled to a reference electrode (WPI, Worcester, MA, USA) using data acquisition software (Biopac Inc., Goleta, CA, USA), as previously published [23]. The selectivity of the electrode for Ca^++^ over other cations, such as Mg^2+^, K^+^, and Na^+^, was >1 × 10^5^.

Electrodes were calibrated before each experiment using known concentrations of CaCl_2_.

Mitochondria (1 mg protein/mL) were added to the incubation medium under gentle stirring and treated with vehicle (DMSO 1%) or isosteviol (3, 10, or 30 μM). The maximal variation in the Ca^++^ concentration of the medium relative to its accumulation in the mitochondrial matrix was measured. Each result was obtained with mitochondria isolated from the hearts of at least three different animals.

##### Measurement of Isosteviol Effects on Mitochondrial Membrane Potential

Potentiometric approach: Mitochondrial membrane potential (Δ*ψ*) was potentiometrically measured with tetraPP-sensitive mini-electrodes coupled to a reference electrode (WPI, Worcester, MA, USA) using a data acquisition software (Biopac Inc.), as described in Calderone et al. [23].

Electrodes were calibrated before each experiment using known concentrations of tetraPP. Mitochondria (1 mg protein/mL) were suspended in the incubation medium under gentle stirring (composition: 120 mM KCl, 5 mM K_2_HPO_4_, 10 mM 4-(2-hydroxyethyl)-1-piperazineethanesulfonic acid (HEPES), 10 mM succinic acid, 2 mM MgCl_2_, and 1 mM EGTA (pH 7.4 adjusted with KOH)). The value of the measured membrane potential was calculated according to the following Nernst-derived experimental equation, as described in a previous paper [18].

(1) $$\Delta \Psi =60\times Log\frac{V_{0}\cdot \frac{{\left[TPP^{+}\right]}_{0}}{{\left[TPP^{+}\right]}_{t}}-V_{t}-K_{0}P}{V_{m}P+K_{i}P}$$

In the equation, the mitochondrial membrane potential (mV) is Δ*ψ*; *V*_0_ is the volume of the incubation medium before the addition of mitochondria; *V_t_* is the volume of the incubation medium after the addition of mitochondria; *V_m_* is the volume of the mitochondrial matrix (mg/mL protein); [*TPP*^+^]_0_ and [*TPP*^+^]*_t_* are the concentrations of *TPP*^+^ before and after the addition of mitochondria at time t, respectively; *P* is the protein concentration (mg); and *K*_0_ and *K_i_* are the apparent external and internal partition coefficients of *TPP*^+^, which were estimated as 14.3 and 7.9 mL/mg, respectively. The concentration of mitochondria was 1 mg of protein/mL [26]. The volume of the mitochondrial suspension was 1 mL.

Changes in the Δ*ψ* were continuously recorded before and after the addition of cumulatively increasing concentrations of isosteviol (range 0.1–30 μM) to the incubation medium.

Each concentration–response curve was obtained with mitochondria isolated from the hearts of at least six different animals.

Spectrofluorimetric approach: The highly sensitive potentiometric approach could not be used for testing the triPP-conjugates of isosteviol due to predicted interference with the tetraPP-sensitive electrode; thus, another experimental method was used. Rhodamine 123 (5 μM) was added to the mitochondrial (1 mg protein/mL) suspension incubation medium (composition: 120 mM KCl, 5 mM K_2_HPO_4_, 10 mM HEPES, 10 mM succinic acid, 2 mM MgCl_2_, and 1 mM EGTA (pH 7.4 adjusted with KOH)) under gentle stirring, and after a baseline recording, the mitochondria were treated with isosteviol, triPP-derivatives (**1**–**5**), or vehicle (DMSO 1%).

The increase in fluorescence (due to the eventual depolarization induced by the tested compounds) was monitored (λ_ex_ = 490 nm, λ_em_ = 535 nm) with an EnSpire multiplate reader (PerkinElmer, Waltham, MA, USA) for 6 min in the dark. Fluorescence values were expressed as the percentage of fluorescence emitted by the rhodamine 123 solution (5 μM) normalized to the fluorescence without mitochondria; the fluorescence value without mitochondria present was set at the maximum value (100%). These experiments were completed within 1.5 h of the mitochondrial isolation step. Each result was obtained with mitochondria isolated from the hearts of three to six different animals.

##### Measurement of triPP-Conjugated Isosteviol Derivative Entry into Mitochondrial Matrix

To verify the accumulation of triPP-conjugated compounds in the mitochondria, the original approach using a tetraPP-sensitive electrode was employed. The addition of compounds **1** through **5** (5 μM) to the suspension buffer was potentiometrically recorded; the addition of mitochondria to the buffer caused a decrease (measured as a reduction in the fluorescence signal) in the extra-mitochondrial concentration of the isosteviol derivatives due to their uptake into the mitochondrial matrix. The amount of loaded compound was quantified by measuring the signal deflection and was expressed as a percentage of the value recorded before the addition of mitochondria.

#### 4.2.2. H9c2 Cell Culture

H9c2 cells (ATTC, Manassas, VA, USA), a subclonal cell line from embryonic rat hearts, were cultured in DMEM/F12 (Sigma-Aldrich, St. Louis, MO, USA) supplemented with 10% of fetal bovine serum (FBS, Sigma–Aldrich, St. Louis, MO, USA), 1.35 g/L glucose, 0.30 g/L sodium bicarbonate, 1 mM sodium pyruvate, 4 mM l-glutamine, 100 units/mL penicillin, and 100 units/mL streptomycin and incubated at 37 °C in a humidified atmosphere of 5% CO_2_.

##### Analysis of ΔΨ Changes on Intact H9c2 Cells

Changes in Δ*ψ* were assessed using the Muse MitoPotential Assay (MCH 100110, Merck Millipore; Darmstadt, Germany), which enables the simultaneous measurement of live, depolarized, depolarized/dead, and dead cells within the same sample. H9c2 cells were treated with DMSO (0.3%, control sample), compound **5** (1 μM), or isosteviol (30 μM) for 2.5 or 5 h. The uncoupling agent CCCP (30 μM) was used as a positive control. To perform the assay, cells were harvested, and the cell pellets were suspended in assay buffer (10^5^ cells/100 μL buffer). According to the manufacturer’s instructions, the MitoPotential working solution was added, and the cell suspensions were incubated at 37 °C for 20 min. After the addition of the Muse MitoPotential 7-AAD dye and incubation at room temperature for 5 min, the variations in ΔΨ were assessed by flow cytometry (Muse Cell Analyzer, Merck Millipore; Darmstadt, Germany). The percentages of depolarized living and depolarized dead cells were analyzed relative to the control and CCCP-treated cells.

#### 4.2.3. In Vivo Model of Acute Infarct of Myocardium

Male Wistar rats were divided into five groups (*n* = 6 in each group): a sham-operated (SHAM) group and four I/R groups. The I/R groups included vehicle (VEHICLE, DMSO 0.1% *v*/*v*), isosteviol 5 μg/Kg (ISO 5), isosteviol 500 μg/Kg (ISO 500), and compound **5** 12.1 μg/Kg groups (compound **5**). This dose of compound **5** (12.1 μg/Kg) is equimolar to isosteviol 5 μg/Kg. Isosteviol and compound **5** were dissolved in DMSO (100 mM) and then diluted in saline solution and injected intravenously 10 min prior to occluding the left coronary artery.

The surgery was performed as previously described [18,23] with minor modifications. Briefly, rats were anesthetized with an intra-peritoneal administration of sodium pentobarbital (70 mg/kg). The trachea was cannulated and connected to a mechanical respiratory apparatus (mod. 7025 Ugo Basile, Comerio, Italy) with a respiratory rate of 70 strokes/min (stroke volume, 1 mL/100 g body weight).

The chest was opened by a left thoracotomy, then a 6–0 surgical needle was passed 2 mm under the left anterior descending coronary artery. A small plastic tube was placed in front of the coronary artery, then the snare was pulled and fixed in place by clamping the tube with a hemostat, inducing a local ischaemia in the myocardium for 30 min (ischaemic period). At the end of the ischaemic period, the plastic tube was removed from the ligature and the artery was re-opened, allowing reperfusion for 120 min (reperfusion period). During monitoring with an electrocardiographic analyzer (Mindray, PM5000, 2 Biological Instruments, Varese, Italy), increases in T wave and ST segments were observed in the ischaemic period; the ST segments returned to normal level during the reperfusion period.

After 120 min of reperfusion period, rats were euthanized by an overdose of pentobarbital sodium and heart was removed, mounted on a Langendorff apparatus, and perfused for 10 min with Krebs solution at 37 °C to wash out the coronary blood vessels. Then, the hearts were deprived of the atria and right ventricle. The left ventricular tissue was dried, frozen at −20 °C for 50 min, cut into 2.0 mm sagittal sections, and immersed in 1% 2,3,5-triphenyltetrazolium chloride (TTC, Sigma-Aldrich) solution at 37 °C for 20 min. Finally, the slices were fixed overnight in 10% formaldehyde and the next day they were photographed. TTC reacts with NADH in the presence of dehydrogenase enzymes to form a formazan derivative, which causes the staining of viable cells in an intense red color. Red-stained viable tissue was easily distinguished from the infracted white-unstained necrotic tissue. The infarct area (Ai) was planimetrically evaluated using an image analyzer program (The GIMP 2). The infarct size was calculated as a percentage of the whole area of the left ventricle (Ai/ALV%).

### 4.3. Data Analysis

All data are expressed as the means ± standard error of the mean. Concentration response curves were analyzed by nonlinear, fitting equations with computer software (GraphPad Prism 4.0, La Jolla, CA, USA). Data were statistically analyzed by ANOVA, and *p* values less than 0.05 were considered indicative of significant differences.

## Figures and Tables

**Table 1:** Potency (pEC50) and efficacy (Emax %) indices measured for isosteviol and its triPP-conjugated derivatives (cmpds). Full = 100%.

Compounds	pEC50	Emax (%)
Isosteviol	3.59 ± 0.074	58 ± 7
Cmpd **1**	5.65 ± 1.67	Full
Cmpd **2**	4.87 ± 0.65	Full
Cmpd **3**	3.85 ± 0.090	82 ± 9
Cmpd **4**	5.01 ± 0.095	Full
Cmpd **5**	5.36 ± 0.060	Full

## Supplementary Materials

Supplementary materials can be found at www.mdpi.com/1422-0067/18/10/2060/s1.
