# Supplementary material for: Mitochondriotropic and Cardioprotective Effects of Triphenylphosphonium-Conjugated Derivatives of the Diterpenoid Isosteviol
Source: Int J Mol Sci. 2017 Sep 26;18(10):2060. doi: 10.3390/ijms18102060 (PMC5666742; doi:10.3390/ijms18102060)
Supplement: Supplementary file 1 [file ijms-18-02060-s001.pdf]

Supplementary Materials: Mitochondriotropic and Cardioprotective Effects of Triphenylphosphonium-Conjugated Derivatives of the Diterpenoid Isosteviol

Lara Testai, Irina Strobykina, Victor V. Semenov, Marina Semenova, Eleonora Da Pozzo, Alma Martelli, Valentina Citi, Claudia Martini, Maria C. Breschi, Vladimir E. Kataev and Vincenzo Calderone

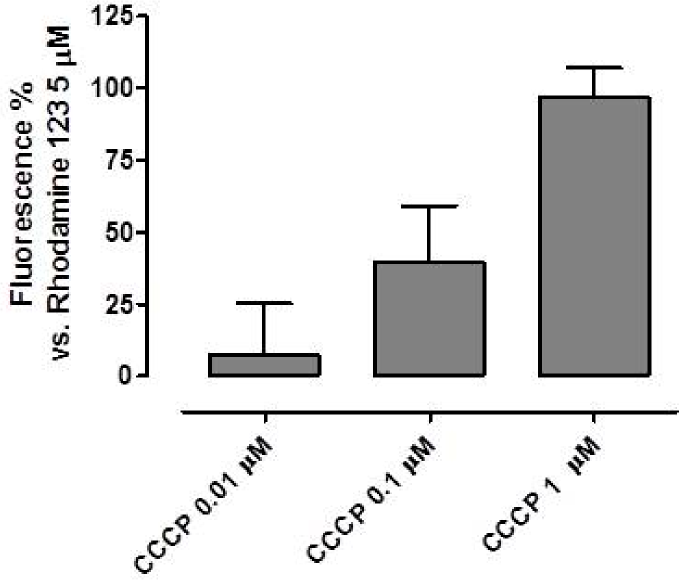

Figure S1. Changes of mitochondrial membrane potential (spectrofluorimetrically measured) following addition at cardiac mitochondria suspension (0.5 mg/ml). Increase (%) in the rhodamine123-induced fluorescence after cumulative addition of the uncoupling agent CCCP.
